# Oxygen in Therapeutics

**Published:** 1886-05

**Authors:** 


### OXYGIN IN THERAPERTES.

Dr. Vanderbeck of Philadelphia, Lectures of Hygiene in the medicochiruragical college, gives a large number of cases in which the use of Oxygen has been proved successful, we give some of them that may be useful to others besides the medical faculty.

**Croup.**—As early as 1794 oxygen had been employed in croup. Bretonneau reported to the Parisian Academy of Medicine, in 1821, good effects in a case of croup. A number of reports are to the effect that the gas is indicated in the suffocating paroxysms of croup, to guard the patient from the consequences of such attacks, which frequently prove fatal, and to place him in such a position as to enable us to apply other remedies, having in such measure the same effect as tracheotomy.

Dr. Pepper says of oxygen in croup: "The inhalation of oxygen has proven rather advantageous in my hands in a few instances." A case in point was dyspnœa, cyanosis, and convulsions, produced by the invasion of the bronchi with the pseudo-membranous process.

Dr. R. H. Goolden, Lancet, October 29, 1879, reports a case of phagedenic ulceration of the pharynx treated by oxygen inhalations with rapid improvement. In the same journal, January, 1880 the Doctor reports favorably of the direct application of oxygen to ulcerating surfaces.

Demarquay says when oxygen is placed in contact with suppurating wounds the discharge lessens and becomes thinner, and granulations quickly spring up.

**Cholera.**—Dr. Smythers used oxygen in this disease with success in the epidemic of 1832. Favorable reports are also given in the Madras army from 1828 to 1844; in india, 1850; in London, 1853; and by Schnarz in 1831. Fay, in Poland, 1831.

**Diabetes.**—Demarquay, Bouchardat, and Simonsin have seen marked diminution in the amount of sugar excreted under oxygen treatment.

**Hydrophobia.**—In the Medical and Surgical Reporter for December 10, 1876, is a translation from a German medical journal of a case of hydrophobia, on the authority of Schmidt and Lebeden, in which the inhalation of three cubic feet of oxygen produced immediate amelioration of the symptoms, and within two and a half hours apparantly restored her to her former condition of health, The next day a more severe attack occured, but was again relieved in forty-five minutes by the inhalation of oxygen. Slight symptoms were thus treated for ten days. The final result was complete recovery.

**Local Use.**—Ringer says: "This gas is useful as a local application to atonic, painful sores, but produces no effect on healthy sores. In senile gangrene, administered as a gaseous bath, for an hour or longer at a time, and repeated six or eight times a day, it is said to be of the greatest use. The livid red changes to a rose color, warmth returns to the tissues, sensation is restored, pain mitigated, and the disease is checked and even sometimes cured."

Dr. A. M—, æt. 55. Came to me suffering from aggravated dyspepsia, with persistent constipation and distressing insomnia. Weight, one hundred and thirty pounds. Has had inflammatory rheumatism, and one sister died of phthisis. Was placed on active oxygen tneatment with nourishing diet—no drugs whatever—and in three weeks gained five pounds in weight, and had an excellent appetite. Constipation relieved and digestion good. Sleeps soundly and has climbed to the summit of a neighboring mountain, nearly five thousand feet above the sea-level. Is to continue the treatment at home.

S. A. C—, merchant, æt. 59. Case of chronic rheumatism, severe dyspepsia, and slowly progressive locomotor ataxia. Oxygen treatment undertaken by means of a portable rubber bag, patient being confined to his bed, and completely helpless. Result of six weeks' treatment, gradual amelioration of all the symptoms. Loco motion became possible by the aid of a cane, and so remains at this writing.

Mrs. S. A. M—, æt. 26. Disease: severe, chronic, gastric catarrh. Completely recovery, and a gain of fifteen pounds in body-weight in six weeks.

In the severer forms of the indigestions the results have been scarcely less brilliant. Scanty or suppressed secretions are gradually restored, absorption and assimilation resume normal activity, and the functions of depuration and defecation, long deranged, obstructed, or interrupted, are surely, normally, and, in a medical sense, permanently restored.

One strange and unexpected phenomenon which accompanies the inspiration of oxygen is the production within the chest of an agreeable sensation of coolness by each inspiration of the gas. The pulse being eighty-four on the 30th of April, when the patient began to inhale the ten litres of gas, had fallen to seventy-six by the time the inhalation was completed, and remained at that point during the remainder of the hospital visit. The pulse becomes thready after three inspirations of oxygen, and so continues for two or three minutes of the duration of the operation.

